# ZFP91 promotes cell proliferation and inhibits cell apoptosis in AML via inhibiting the proteasome-dependent degradation of RIP1

**DOI:** 10.7150/ijms.67436

**Published:** 2022-01-01

**Authors:** Zhonghui Zhang, Liang Zhong, Wenran Dan, Xuan Chu, Chen Liu, Xu Luo, Peng Wan, Zhenyan Liu, Yang Lu, Xiao Wang, Beizhong Liu

**Affiliations:** 1 Central Laboratory of Yongchuan Hospital, Chongqing Medical University, Chongqing 402160, China; 2 Key Laboratory of Laboratory Medical Diagnostics, Ministry of Education, Department of Laboratory Medicine, Chongqing Medical University, Chongqing 400016, China

**Keywords:** ZFP91, RIP1, E3 ligase, Ubiquitination, AML, Proteasome degradation

## Abstract

Acute myeloid leukemia (AML) is a quickly progressive and devastated hematological malignancy with large rate of relapse and the appearance of chemotherapy resistance. Therefore, the identification of new therapeutic targets is urgent. ZFP91 is a hidden oncogene. Nevertheless, how ZFP91 takes part in regulating AML is less clear. Our research aims at investigating the molecular mechanisms and uncovering the effects of ZFP91 on AML. This research demonstrates that ZFP91 boosts AML cell proliferation and stops AML cell apoptosis. Mechanistically, experimental results showed the interaction between ZFP91 and RIP1 and inhibitory effect of ZFP91 on the K48-linked ubiquitination of endogenous RIP1, which is an important molecule in AML. Taken together, our results provide the evidence that targeted inhibition of ZFP91 could be a hopeful measure to treat AML.

## Introduction

As the most usual form of adult acute leukemia, acute myeloid leukemia (AML) has the characteristics such as infiltering the bone marrow, blood, and other tissues by abnormally differentiated, clonal, proliferative, and sometimes weakly differentiated cells of the hematopoietic system 1-3. Its incidence is increasing, and the overall long-term survival rate is low due to the high recurrence rate of the disease after standard chemotherapy 4. In the past few years, with multiple new FDA-approved drugs, the prospects for AML treatment have changed greatly, However, the outcomes of most AML patients are still frustrating 5,6. Hence, the hidden mechanism in charge of AML tumorigenesis shall be explored for the identification of extra hopeful targets for AML therapy.

Ubiquitination importantly modifies proteins in eukaryotic cells; ubiquitination is a core mediator for different intracellular signaling activities including kinase activation, proteasomal degradation, subcellular localization, and endocytosis 7,8. The ubiquitin-proteasome system (UPS) exerts a core effect on cancer tumorigenesis and progression. UPS components such as 3 enzyme families (E1, E2, and E3) and proteasomes, are promising therapeutic targets for cancer and other diseases 9-11. As everyone knows, E3 ubiquitin ligases exert an important effect on regulating cell proliferation, the cell cycle and cell apoptosis 12. There are 3 thalidomide, lenalidomide, and pomalidomide, E3-targeting small-molecule drugs approved by the FDA; moreover, PROTAC technology has now made it possible to target protein degradation 13,14. Taken together, these studies suggest that targeting E3 ubiquitin ligases is a promising cancer treatment strategy.

As a member of the zinc finger protein family with 5 zinc finger domains, ZFP91 is a new E3 ubiquitin ligase 15. ZFP91 exerts an important effect on tumorigenesis and takes part in various oncogenic pathways such as β-catenin, NF-κB, MAPK, and HIF-1α 16-19. The study of ZFP91 in regulating biological function and its effects on tumorigenesis can make contributions to developing given inhibitors to enhance method to treat AML. In a recent study, it was shown that XD2-149, a proteolysis-targeting chimera (PROTAC) based on napabucasin, degrades ZFP91 with DC50 values in the nanomolar scope 20. According to these outcomes, ZFP91 could be a promising therapeutic target for AML.

As a 76kDa protein, receptor-interacting serine/threonine-protein kinase 1 (RIP1) mainly regulates the cellular decision in the activation of cell death and NF-κB signaling. It responds to a series of inflammatory and pro-death responses in diseases 21-23. Targeting RIP1 pathways has been proved to be a hopeful therapeutic chance for AML cancer cells harboring the FLT3-ITD mutation 24. Moreover, our ongoing research shows that RIP1 could promote the occurrence and development of AML. Evidence for the important role of RIP1 in disease has contributed to inhibiting RIP1 as a feasible therapeutic strategy in AML. Nevertheless, the administrative mechanism of RIP1 in AML is unclear.

This research presents several pieces of evidence that ZFP91 promotes the initiation and development of AML via interacting with RIP1 and inhibiting the K48-linked ubiquitination of RIP1. Our study better understands the molecular mechanism of AML pathogenesis and provides a theoretical foundation for identifying ZFP91 as a hidden therapeutic objective in AML.

## Materials and Methods

### Cell culture

The Cell Bank of the Chinese Academy of Sciences (Shanghai, China) provided human myeloid cell lines such as KG1a, THP1, NB4, and U937. The culture of all cells was performed in RPMI-1640 medium (Thermo Fisher Scientific, Waltham, MA, USA) with 10% of fetal bovine serum (FBS, Thermo Fisher Scientific) and 1% of penicillin/streptomycin solution (Beyotime, Shanghai, China) in a context with 5% CO2 at 37 °C.

### CCK8

A Cell Counting Kit-8 (no. HY-K0301; MCE, USA) was employed to detect the proliferation rates. The seeding of logarithmically growing cells into 96-well plates was conducted at a density of 5 × 10^3^ cells per well. At the displayed time points after incubation, the cell culture medium was added with 10 μl of CCK-8 solution. Optical density (OD) was measured after 2-hour incubation. A microplate reader (Bio Tek, CA, USA) was performed to measure the absorbance at 450 nm.

### Growth curve analysis

The seeding cells was conducted in 6-well plates at a density of 3 × 10^5^ cells per well for growth curve analysis. Next to overnight incubation, the culture medium was abandoned and substituted with fresh medium. Cells were counted with a hemocytometer (Nikon, Tokyo, Japan) 0, 24, 48, 72, and 96 h after altering the medium.

### Western blotting

Ice-cold phosphate-buffered saline (PBS) was employed to wash the cells in every group for 3 times, and ice-cold radioimmunoprecipitation assay (RIPA) lysis buffer (no. P0013; Beyotime, China) with protease inhibitor phenyl methane sulfonyl fluoride (PMSF) (Beyotime Biotechnology, Shanghai, China) (1:100) was adopted to extract the total protein. The BCA method was adopted to measure protein concentration with a BCA protein assay kit (Beyotime Biotechnology). Equal amounts of protein was added in 10%-15% sodium dodecyl sulfate-polyacrylamide gel, which was next to by transfer onto polyvinylidene fluoride membranes (Millipore, MA, USA). The 1-hour blocking of membranes was performed with 5% non-fat milk (Boster Biological Technology, Wuhan, China), which was next to overnight incubation with primary antibodies at 4 °C. Applied primary antibodies were shown below: ZFP91(1:500; Abcam, UK), RIP1(1:1000; Cell Signaling Technology, USA), β-actin(1:1000; Boster Biological Technology, USA), Tubulin(1:1000; Cell Signaling Technology, USA), Histone-H3(1:1000; Cell Signaling Technology, USA), cl-PARP(1:1000; Bimake, USA), BAX(1:1000; Bimake, USA), Bcl-2(1:1000; Bimake, USA), c-myc(1:1000; Cell Signaling Technology, USA), β-catenin(1:1000; Cell Signaling Technology, USA), GSK3-β(1:1000; Cell Signaling Technology, USA), p-GSK3-β(1:1000; Cell Signaling Technology, USA), NF-κB(1;1000; Abmart, China), ZFP91(1:1000; Santa Cruz Biotechnology, USA), NIK(1:1000; Santa Cruz Biotechnology, USA), caspase8(1;1000; Wanleibio, China), TRAF2(1:1000; Bimake, USA), K48-linkage Specific Polyubiquitin (1:1000; Cell Signaling Technology, USA), Ub(1:1000; Santa Cruz Biotechnology, USA), and IgG(1:1000; Cell Signaling Technology, USA). The 1-hour incubation of membranes was conducted with secondary antibodies (1:4000; Biosharp, China) at room temperature. A strengthened chemiluminescence (ECL) Ultra Western HRP Substrate kit (WBUL S0100; EMD Millipore, USA) was adopted to detect the protein signals with an ECL visualization system (GE Healthcare, USA).

### qRT-PCR

The extraction of total RNA was conducted with TRIzol reagent (Takara, Japan), which was followed by transcribing to cDNA with a PrimeScript RT regent Kit (Takara, Tokyo, Japan). qRT-PCR on a CFX Connect real-time PCR operating system (Bio-Rad, USA) was performed with SYBR Premix Ex Taq II kit (Takara, Japan). The synthetization of Primers was made at Sangon Biotech (Shanghai, China). Table 1 shows the orders of primers applied for qRT-PCR.

### Cell infection

The recombinant lentivirus interfering with ZFP91 expression was obtained from Genechem (Shanghai, China). The 48-hour infection of AML cells was with shRNA lentivirus with infection enhancer and then subjected to 2μg/mL puromycin (Genechem, Shanghai, China) selection for a week. Subsequent experiments adopted stably transfected cells.

### Cell transfection

The cells were transfected with empty vector and ZFP91 overexpression plasmids (Genechem, Shanghai, China) using transfection reagent (Invitrogen, Carlsbad, CA, USA) according to the manufacturer's protocol. After 48 hours of transfection, the cells were collected for western blotting, ubiquitination analysis.

### Co-IP

Co-IP was conducted on basis of the producer's guideline. Protein A/G Magnetic Beads (no. HY-K0202; MCE, China) were put into 2 EP tubes, which was followed by twice washes with PBST. Next resuspension in 200μl of binding solution, after 2-hour incubation on an inverted mixer, it was received magnetic isolation and washed twice. Then, 500 μl of protein was put to every tube, which was next to overnight incubation with pre-conjugated antibodies beads on the inverted mixer at 4 °C. Next day, the proteins with the standard protocol were analyzed with western blotting. Specifically, secondary antibody applied to western blotting was IPKine™ HRP, Mouse Anti-Rabbit IgG LCS (1:1000, Abbkine Scientific Co., Ltd) to eliminate heavy chain interference.

### Cycloheximide assay

To investigate whether RIP1 protein stability is regulated by ZFP91, ShControl and shZFP91 cells were handled with 100 μg/mL cycloheximide (CHX; MilliporeSigma) to block de novo protein synthesis for 0, 4, and 8 h. Western blot was used to analyze protein at each time point.

### In vivo ubiquitination assay

The 8-hour treatment of transfected cells was made with 10 μmol/l MG132 (HY-13259, MCE, China) before washing with PBS, which was followed by collection by centrifugation. The overnight incubation of the supernatants of the cell lysates was made with RIP1 antibody and protein A/G at 4 °C. After being washed, the isolation of proteins precipitated was conducted by SDS-PAGE, which was followed by immunoblotting with ubiquitin antibodies.

### Flow cytometry assay

An annexin V/FITC and PI apoptosis detection kit and flow cytometry were adopted to evaluate cell apoptosis. The transfection of cells with shRNA was performed, which was followed by three washes with PBS. An Annexin V FITC-PI apoptosis detection kit (BD Biosciences, NJ, USA) was adopted to perform apoptosis staining on basis of the producer's guideline. A CytoFLEX flow cytometer (Beckman Coulter, USA) was employed to analyze stained cells. CytExpert V2.3.0.84 software (Beckman Coulter, USA) was used for data acquisition. For the assessment of the cell cycle allocation, PBS was adopted to wash the cells transfected with shRNA twice, Then, the overnight fixing of cells was performed with 75% ethanol (absolute ethanol/PBS ratio = 3:1) at -4 °C. After 30-minute incubation with propidium iodide (PI; 100 μg/ml) solution at room temperature (RT) in the dark, a CytoFLEX flow cytometer (Beckman Coulter, USA) was employed to measure the cells. CytExpert V2.3.0.84 software was adopted for the analysis of the cell cycle distribution.

### Statistical analysis

All data originated from 3 independent tests. Information was shown as means ± SD. The calculation of statistical significance was conducted by unpaired t tests or one-way ANOVA with the SPSS (Version 17.0) and GraphPad (Prism 5.0) software programs. P < 0.05 was regarded statistically obvious (* P < 0.05, ** P <0.01, *** P < 0.001).

## Results

### Expression of ZFP91 and the efficiency of knockdown of ZFP91 in AML

**Figure 1:**
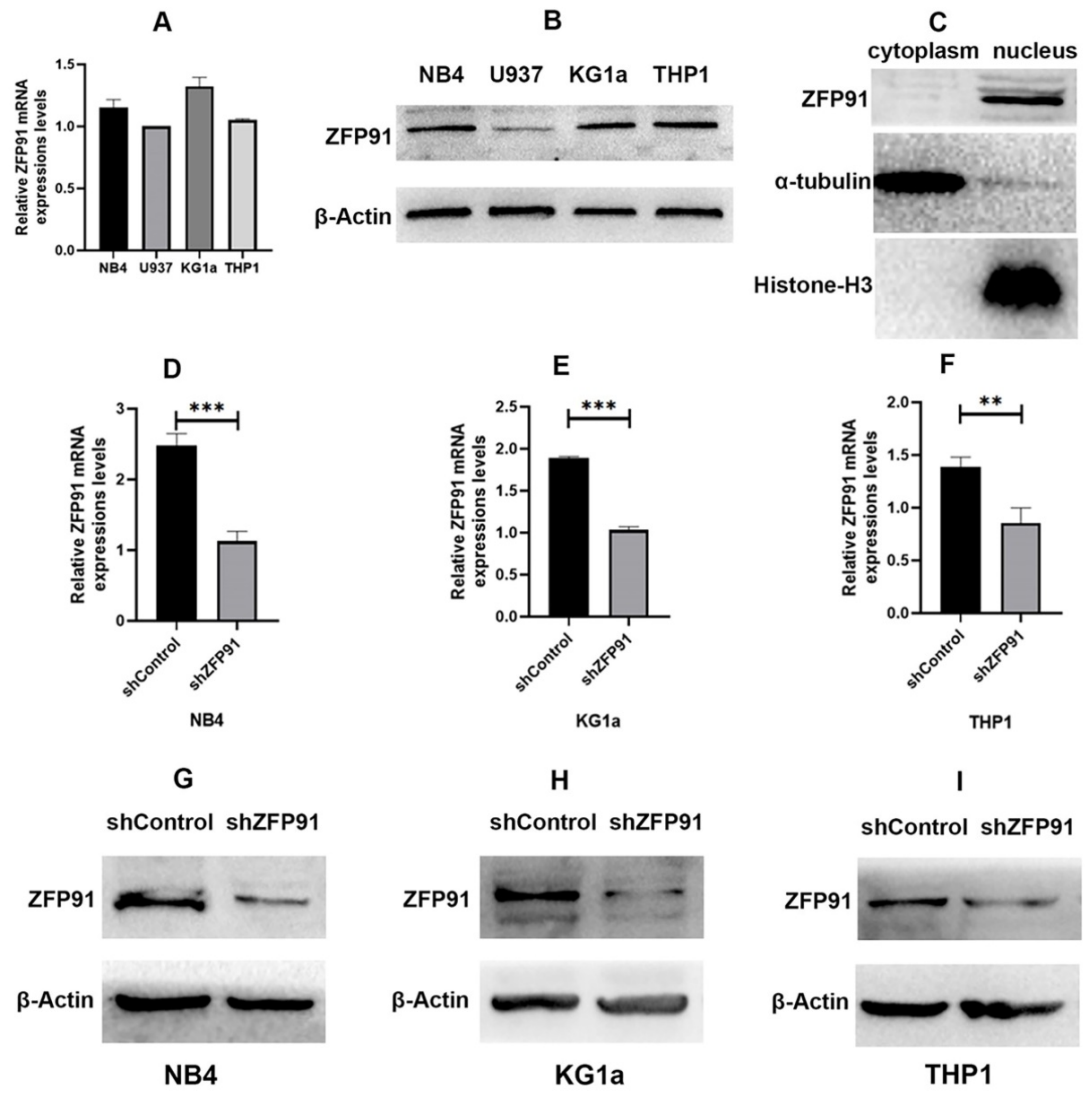
** Expression and knockdown efficiency of ZFP91 in AML cells.** (A) The mRNA levels of ZFP91 in NB4, U937, KG1a and THP1 cells were measured by qRT-PCR. (B) The protein levels of ZFP91 in NB4, U937, KG1a and THP1 cells were detected by western blotting. (C) Nuclear and cytoplasmic protein fractions were collected from NB4 cells, protein concentration was measured by BCA Protein Assay Kit, equal amounts of protein samples were loaded, and the expression of ZFP91 was detected by western blotting. (D-F) qRT-PCR analysis of ZFP91 mRNA levels in the NB4, KG1a and THP1 cells after infecting with shZFP91 lentivirus. (G-I) Western blotting analysis of ZFP91 protein levels in the NB4, KG1a and THP1 cells after infecting with shZFP91 lentivirus.

It is reported that ZFP91 is overexpressed in AML and is more likely to exert a significant effect on cell proliferation and apoptosis, and may act as a molecular marker for AML 25,26. ZFP91 seems to be an important molecule in many cancers including AML. First, we detected the mRNA and protein expression extents of ZFP91 in different human AML cell lines such as NB4, U937, KG1a, and THP1 cells (Fig. 1A and B). We found that ZFP91 is widely expressed in the AML cell lines we tested. Then we sought to determine the location of ZFP91 in the cells. α-tubulin, a nuclear reference protein, is mainly expressed in the nucleus, and Histone-H3, a cytoplasmic reference protein, is mainly expressed in the cytoplasm. Meanwhile, ZFP91 is mainly expressed in the nucleus while loading equal protein. As shown in Fig. 1C. We found that ZFP91 is located in the nucleus mainly. For assessing whether ZFP91 expression exerts a significant effect on AML progression, lentivirus was adopted for the knockdown of ZFP91 in NB4, KG1a, and THP1 cells, and we determined efficient ZFP91 knockdown at the mRNA (Fig. 1D, E, and F) and protein level (Fig. 1G, H, and I).

### ZFP91 induces AML cell proliferation

**Figure 2:**
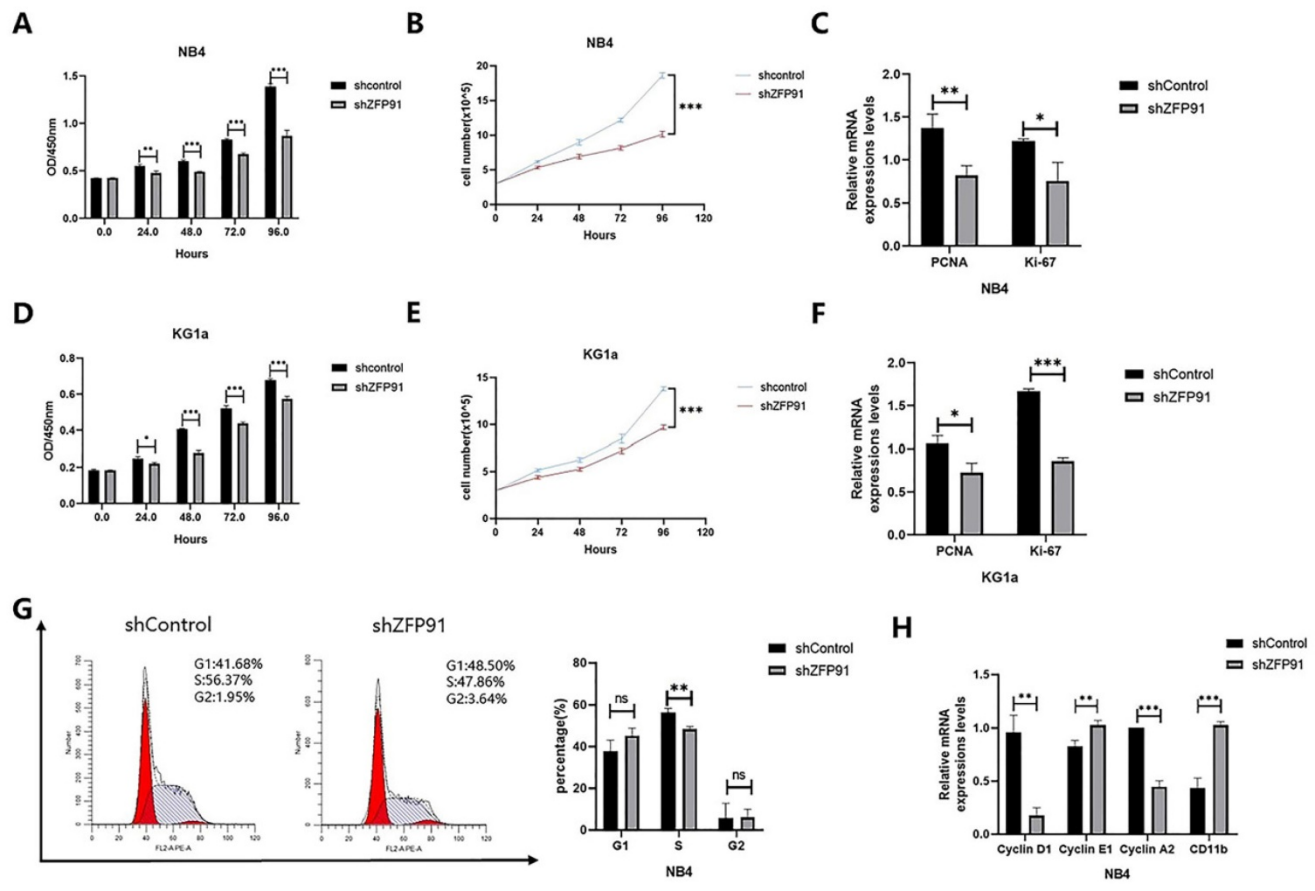
** Knockdown of ZFP91 expression inhibits AML cell proliferation.** (A, D) Cell viability was analyzed by the CCK-8 assay in NB4(A) and KG1a(D) cells. (B, E) Cell viability was analyzed by the cell counting assay in NB4(B) and KG1a (E) cells. (C, F) The mRNA levels of PCNA and Ki-67 were determined by qRT-PCR in NB4(C) and KG1a(F) cells. (G) Cell cycle was determined by flow cytometry. (H) The mRNA levels of CyclinD1, Cyclin E1, Cyclin A2 and CD11b were detected by qRT-PCR.

Given that ZFP91 is a tumor promotor in several cancers, we wished to explore whether ZFP91 can affect AML cell propagation and cell cycle allocation. Cell proliferation was monitored by using a CCK-8 assay and cell growth curve. Our results showed that knockdown of ZFP91 expression inhibited NB4 and KG1a cells' proliferation (Fig. 2A, B, D, and E). The expression of both propagation markers PCNA and Ki-67 was downregulated due to the knockdown of ZFP91 (Fig. 2C and F). The accurate transition from G1 to S phase is important for the control of cell proliferation, and its misregulation may promote oncogenesis 27. To examine the role of ZFP91 in the cell cycle, we adopted flow cytometry to evaluate the distribution of the NB4 cell phase. According to Fig. 2G, the knockdown of ZFP91 decreased the percentage of S phase cells. It is well-known that the unrestrained progress into S phase is a hallmark of cancer. In addition, we examined the effects of ZFP91 on Cyclin D1, Cyclin E1, Cyclin A2, and CD11b, the core regulators of the cell cycle. Cyclin D1, an important cell cycle protein promoting the cell cycle from G1 to S period, was downregulated in ZFP91 knockdown cells by comparing it with the control cells. As a result of the decisive role Cyclin A2/E1 play in the cell cycle, Cyclin E1 was upregulated in ZFP91 knockdown cells, and Cyclin A2 was downregulated in ZFP91 knockdown cells. CD11b, as an index of cell differentiation, was upregulated in ZFP91 knockdown cells (Fig. 2H). According to these outcomes, ZFP91 knockdown stops the proliferation of AML cells and the effect on cell proliferation may be due to the arrest of S phase.

### ZFP91 inhibits AML cell apoptosis

**Figure 3:**
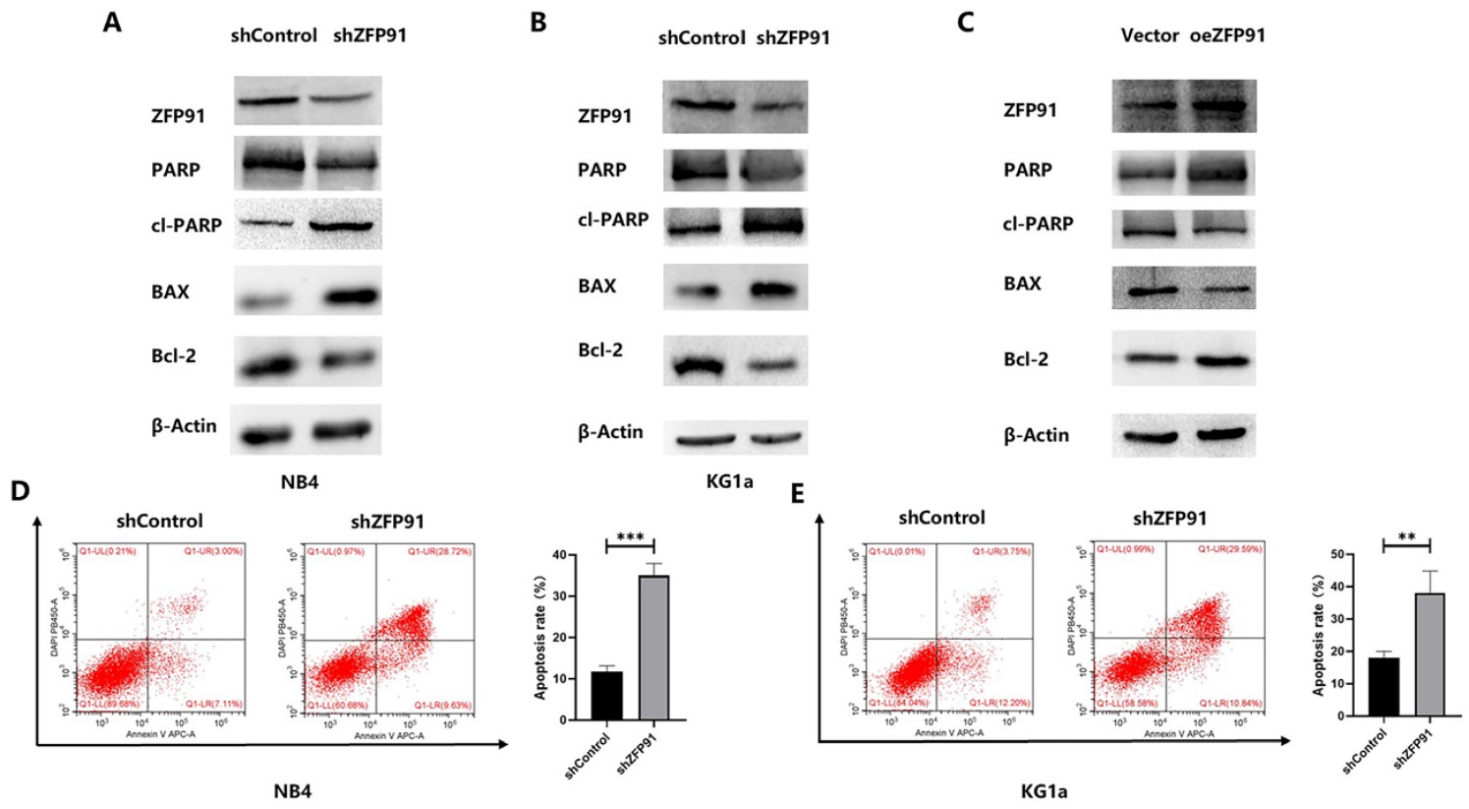
** Knockdown of ZFP91 expression induced AML apoptosis.** (A-C) The levels of the apoptosis-associated proteins PARP, cleaved PARP, BAX, Bcl-2 in NB4 and KG1a cells were analyzed by western blotting. (D, E) The apoptosis of NB4 and KG1a cells was evaluated by flow cytometry using an annexin V/PI detection kit.

Then, the role of ZFP91 downregulation in AML cell apoptosis was examined. Western blotting and flow cytometry were employed to assess some apoptotic parameters. The western blotting outcomes demonstrated that the knockdown of ZFP91 greatly grew the extents of the pro-apoptotic proteins cleaved PARP and BAX, while greatly decreasing the level of the anti-apoptotic protein PARP and Bcl-2 in NB4 and KG1a cells (Fig. 3A and B). The opposite western blotting results were obtained in KG1a cells overexpressing ZFP91 by plasmid transfection (Fig. 3C). According to the flow cytometric information, the apoptotic rate of NB4 and KG1a cells in the ZFP91 knockdown group was greatly added by comparing with the control group (Fig. 3D and E).

### RIP1 exerts a significant effect on the NF-κB signaling pathway via interacting with NIK

**Figure 4:**
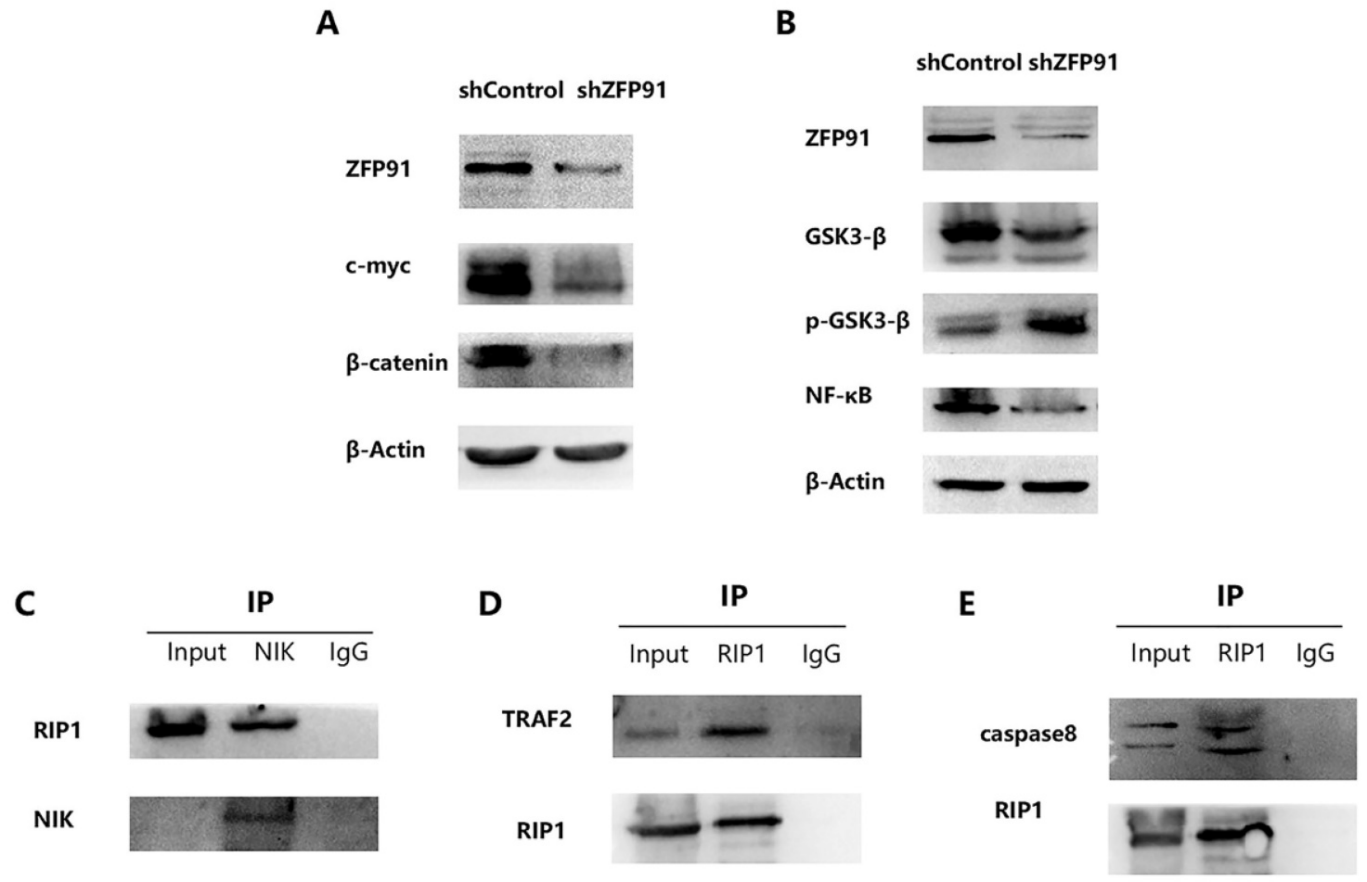
** RIP1 plays an important role in NF-κB Signaling pathway via interacting with NIK.** (A) The protein expression levels of c-myc and β‐catenin was determined by western blotting. (B) The protein expression levels of GSK3-β, p-GSK3-β and NF-κB was detected by western blotting. (C) Protein extracted from NB4 cells was co-immunoprecipitated with anti-NIK antibody and blotted with anti-RIP1 and anti-NIK antibodies. (D) Protein extracted from NB4 cells was co-immunoprecipitated with anti-RIP1 antibody and blotted with anti-TRAF2 and anti-RIP1 antibodies. (E) Protein extracted from NB4 cells was co-immunoprecipitated with anti-RIP1 antibody and blotted with anti-caspase8 and anti-RIP1 antibodies.

It has been reported that ZFP91 possesses cancer-promoting functions. And it has been displayed that ZFP91 promotes pancreatic cancer propagation, move, and invasion by the activation of β-catenin signaling 28. It has been proved that β-catenin and c-myc are typical cancer-promoting molecules in many cancers including AML. So, β-catenin and c-myc expression in NB4 ZFP91 knockdown cells were examined. According to the western blot analysis, the expression of β-catenin and c-myc were greatly downregulated in NB4 ZFP91 knockdown cells by comparing with control cells (Fig. 4A). This indicated that ZFP91 might exert a significant effect on the development of AML as a cancer-promoting factor. It has been disclosed that ZFP91 boosts prostate cancer propagation via the NF-κB pathway and tumorigenesis of colon cancer by the upregulation of the HIF-1α level 18,29. NF-κB is increasingly considered a crucial molecule in many steps of cancer occurrence and development 30. Given that ZFP91 has an essential effect on NF-κB pathway in other cancers 31,32, we investigated the potential role of ZFP91 in AML. In our results, the protein levels of NF-κB declined significantly in NB4 ZFP91 knockdown cells compared with the control group (Fig. 4B). Meanwhile, we confirmed that GSK-3β has a regulating effect on the NF-κB signaling pathway 33. In addition, GSK3β is generally overexpressed and aberrantly nuclear localized in AML. GSK3-β and p-GSK3-β are considered important regulators of NF-κB and play significant roles in AML development 34,35. On the basis of these studies, we further investigated the impact of ZFP91 knockdown on GSK3-β. In our results, the expression extent of GSK3-β was down-controlled while the expression extent of p-GSK3-β was upregulated after the knockdown of ZFP91 expression (Fig. 4B). Taken together, our results indicate that ZFP91 may have a potential role in the NF-κB signaling pathway in AML. The stabilization and activation of NIK by ZFP91 in a ubiquitination-dependent manner indicates it is a significant regulator of the NF-κB pathway. In addition, NIK, RIP1, TRAF2, and IKKs were considered to exist in a large multiprotein complex termed the signalosome 36-39. The interaction of NIK, TRAF2, and RIP1 in AML cells was detected by Co-IP analysis. Our results suggest that immunoprecipitation with anti-NIK antibody pulled down endogenous RIP1 along with NIK (Fig. 4C), and immunoprecipitation with anti-RIP1 antibody pulled down endogenous TRAF2 along with RIP1 (Fig. 4D). RIP1, as an important molecule in cell death, plays a critical role in cancer development. Our coimmunoprecipitation (Co-IP) assay results suggest that endogenous caspase 8 was detected after being immunoprecipitated with anti-RIP1 antibodies but not with lgG control (Fig. 4E). This suggests its potential role in cell death in AML. Therefore, we focused our research on the effect ZFP91 had on RIP1.

### ZFP91 controls the protein expression of RIP1 with no alteration of the mRNA levels

**Figure 5:**
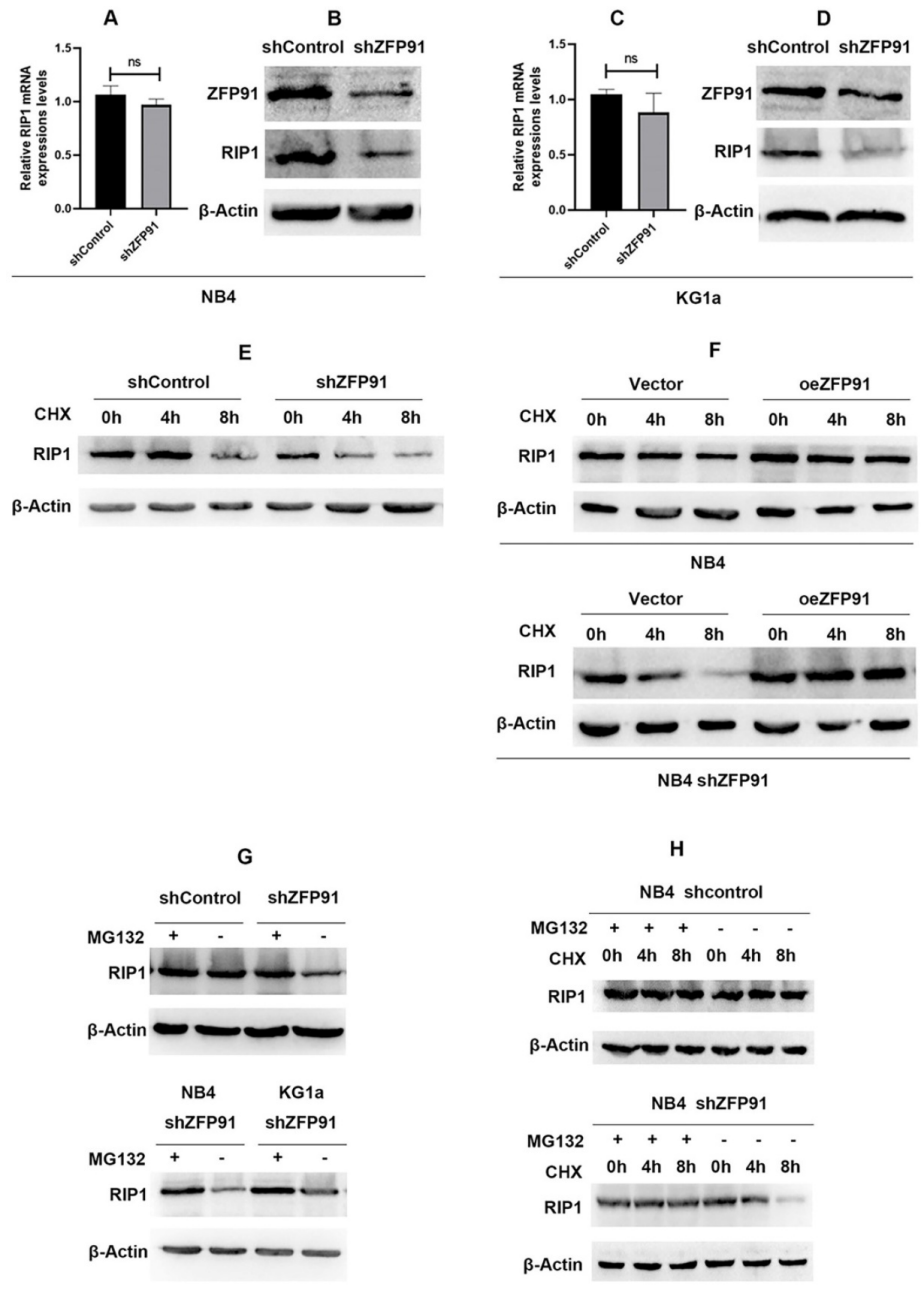
** ZFP91 regulates the protein expression of RIP1 without altering the mRNA levels.** (A, C) The mRNA expression level of RIP1 was analyzed after downregulating ZFP91 by qRT-PCR. (B, D) The protein expression level of RIP1 was detected after downregulating ZFP91 by western blotting. (E) NB4 infecting with shZFP91 lentivirus were incubated with 10 μg/ml cycloheximide (CHX) for various time periods respectively and the expression level of RIP1 was determined by western blotting. (F) Exogenous RIP1 protein levels were determined by western blotting following ZFP91 overexpression in the presence of CHX for the indicated times. (G) NB4 infecting with shZFP91 lentivirus were treated with 10 μmol/l MG132 for the indicated time. The protein level of RIP1 were then examined by immunoblotting analysis. (H) NB4 were treated with 10 μmol/l MG132 and 10 μg/ml cycloheximide (CHX) for the indicated time, the expression level of RIP1 was determined by western blotting.

First, we detected the mRNA expression levels of RIP1 after the knockdown of ZFP91 in NB4 and KG1a leukemia cells. The RT-PCR results indicated that there is no statistical difference between ZFP91 knockdown cells and control cells in mRNA levels (Fig. 5A and C). Then, the WB results suggested that the knockdown of ZFP91 induces the downregulation of RIP1 protein levels (Fig. 5B and D). To study the mechanism behind these results, we further investigated whether ZFP91 is involved in the regulation of RIP1 protein stability. Upon silencing of ZFP91, the degradation of endogenous RIP1 protein was sped up in a time-dependent manner after protein synthesis inhibitors cycloheximide (CHX) treatment (Fig. 5E). Upon overexpression of ZFP91, the degradation of exogenous RIP1 protein was alleviated (Fig. 5F). Notably, this alleviated trend was more obvious in ZFP91 knockdown cells. It shows that the overexpression of ZFP91 is feasible. Therefore, the relatively insignificant trend of the endogenous RIP1's degradation after overexpressing ZFP91 in AML cells may demonstrate the high expression of ZFP91, which resulted in RIP1's stability. Furthermore, the protein expression level of RIP1 was increased after treatment with the proteasome inhibitor MG132 in ZFP91 knockdown cells. This result suggests that proteasome degradation plays an important role in RIP1. Then, we confirmed this result in 2 different ZFP91 knockdown leukemia cells, NB4 and KG1a (Fig. 5G). The expression level of RIP1 protein could be stabilized by MG132 in ZFP91 KD cells compared to the control cells (Fig. 5H), further indicating that the degradation of RIP1 in the leukemia cells depends on the proteasome pathway after the knockdown of ZFP91.

### ZFP91 regulates RIP1 protein stability via inhibiting RIP1 ubiquitination

**Figure 6:**
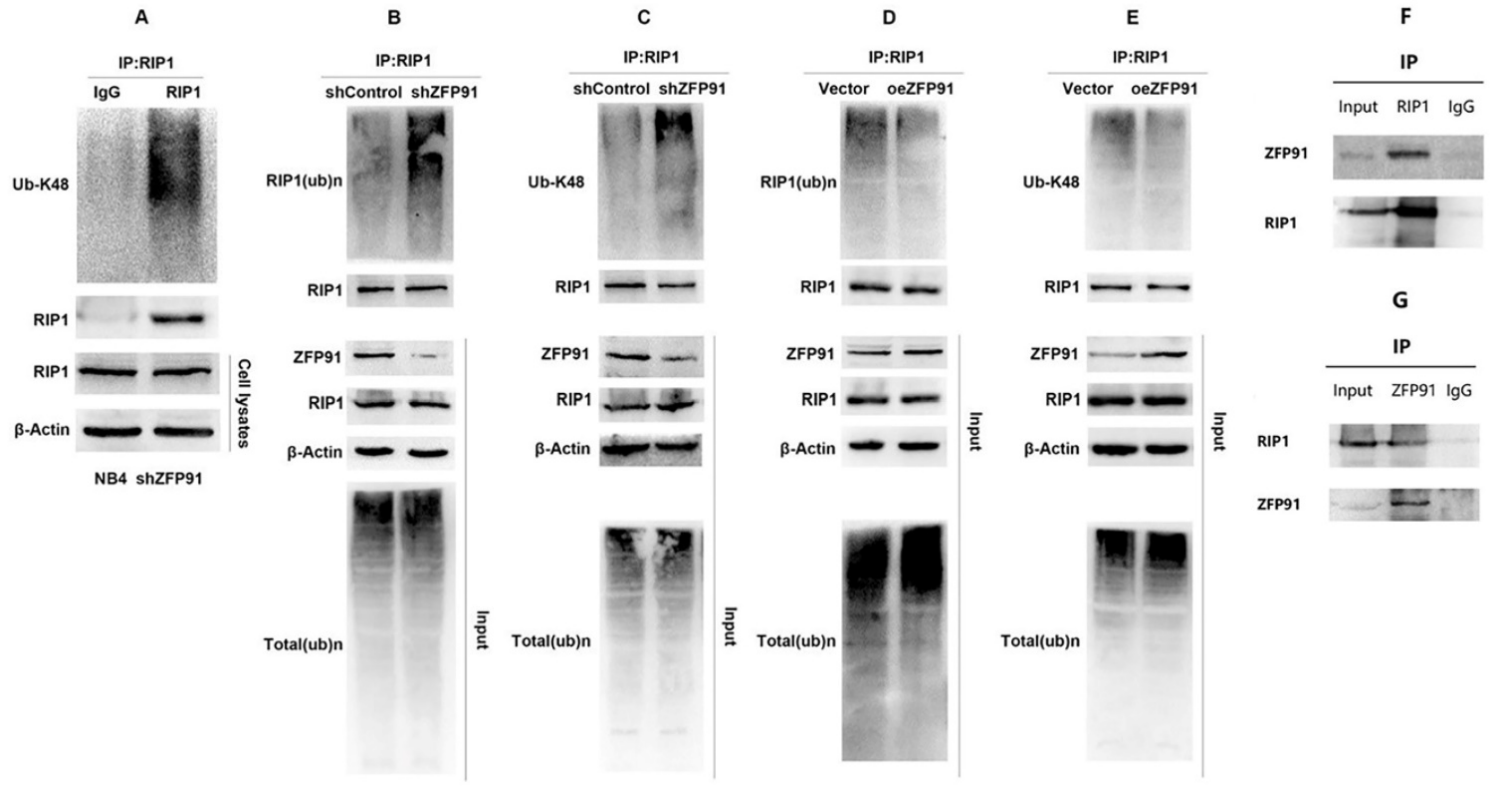
** ZFP91 interacts with RIP1 and Inhibits RIP1 ubiquitination.** (A) The ubiquitination levels of RIP1 in NB4 cell infecting with shZFP91 lentivirus were measured by immunoprecipitation assay using anti-RIP1 antibody and the immunoprecipitates were subjected to western blot with anti-K48-specific ubiquitin antibodies. (B, C) The ubiquitination levels of RIP1 in ZFP91 knockdown cells were measured by vivo ubiquitination assay using anti-RIP1 antibody and the immunoprecipitates were subjected to western blot with anti-Ub (B) and anti-K48-specific(C) ubiquitin antibodies. (D, E) The ubiquitination levels of RIP1 in ZFP91 overexpression cells were measured by vivo ubiquitination assay using anti-RIP1 antibody and the immunoprecipitates were subjected to western blot with anti-Ub (D) and anti-K48-specific(E) ubiquitin antibodies. (F) Protein extracted from NB4 cells was co-immunoprecipitated with anti-RIP1 antibody and blotted with anti-ZFP91 and anti-RIP1 antibodies. (G) Protein extracted from NB4 cells was co-immunoprecipitated with anti-ZFP91 antibody and blotted with anti-RIP1 and anti-ZFP91 antibodies.

As mentioned above, protein degradation mediated by the ubiquitin proteasome pathway is an important mechanism of regulating intracellular protein level and function. K48-linked ubiquitination mediates proteasome degradation of proteins. As shown in Fig. 6A, RIP1 was subjected to K48-linked ubiquitination in NB4 cells. Then we detected the ubiquitination level of RIP1 in NB4 ZFP91 knockdown cells and control cells by vivo ubiquitination assays. Our results suggest that the knockdown of ZFP91 in NB4 cells promoted the ubiquitination of endogenous RIP1 (Fig. 6B). K48-linked ubiquitination was promoted in NB4 ZFP91 knockdown cells, in contrast to control cells as well (Fig. 6C). Conversely, the total ubiquitination level and K48-linked ubiquitination level of endogenous RIP1 was inhibited in ZFP91 overexpression cells (Fig. 6D and E). To explore where there is a correlation between ZFP91 and RIP1, we performed coimmunoprecipitation (Co-IP) assays. The results showed that endogenous ZFP91 was detected after immunoprecipitation with anti-RIP1 antibodies, in contrast to the lgG control (Fig. 6F), and endogenous RIP1 was detected after immunoprecipitation with anti-ZFP91 antibodies, in contrast to the lgG control (Fig. 6G).

## Discussion

AML is the most usual form of acute leukemia among adults, and it is characterized by clonal heterogeneity of hemopoietic progenitor cells 40. Treatment options for AML are still limited, and outcomes are poor. A growing body of evidence has displayed that the dysregulation of ZFP91 is related to cancer growth 41-43. Nevertheless, the accurate molecular mechanisms are not well recognized. Herein, our data demonstrated that ZFP91 acts as a cancer promoter by inhibiting the K48-linked ubiquitination of RIP1, which promotes cell proliferation and suppresses cell apoptosis in AML. This is the first study to prove the functional effect of ZFP91 on the growth of AML, showing that ZFP91 is a hopeful therapeutic target for AML.

More and more studies suggest that ZFP91, a backward-looking 63.5 kDa nuclear protein with structural motifs feature of transcription elements, is an atypical E3 ubiquitin ligase. ZFP91 takes part in different biological processes, and it promotes cancer development by the control of the NF-кB signaling pathway, FOXA1, HIF-1α, and so on 29,31,44. An accumulating body of research indicates that its inhibition or degradation could be effective as an anti-cancer treatment measure.

Past researches have indicated that the expression of ZFP91 grew in AML patients compared to healthy donors. According to these outcomes, ZFP91 may be core to the survival of AML cells. It is likely that ZFP91 exerts a significant effect on cell proliferation and apoptosis as a molecular marker for AML 25. This research displayed that the levels of proliferation were apparently declined and the levels of apoptosis markedly grew in AML cells when downregulating ZFP91, and these results help to elucidate the hidden mechanisms of ZFP91 in AML.

Our outcomes demonstrate that the expression of ZFP91 is positively related to the protein level of RIP1 instead of the mRNA level of RIP1. RIP1 is an important mediator of cell death and inflammation. RIP1 contains an N-terminal kinase domain, a C-terminal death domain, and a medium field with a RHIM (receptor-interacting protein homotypic interacting motif) that can bind to other RHIM-including proteins 45. Complicated ubiquitylation, phosphorylation, and other alternation events on RIP1 determine the activation of RIP1, and the function of RIP1 also depends on post-translational modification. Different post-translational modifications in RIP1 exhibit different or even contrary effects. It has been suggested that ubiquitination could directly influence the kinase activity of RIP1 46. The K63-, K48-, and K11-linked ubiquitin chains are 3 major types of ubiquitination linkages for RIP1 that have been identified by mass spectrometry approaches 47. Thus, it shall be noted that the influence of the polyubiquitination of RIP1 on its expression and transcriptional activity is greatly important. RIP1 can be degraded through K48-linked ubiquitination and can then promote cell death, but there are few studies on this aspect, and further research is needed to prove this point 48. However, most existing research on the modification of RIP1 focuses on the K63-linked ubiquitination. The outcomes hypothesized that ZFP91 can affect the proteasome-dependent degradation of RIP1 and to destabilize its expression by binding to RIP1. Furthermore, given the important role of RIP1 in mediating necroptosis, future research can focus on the role ZFP91 plays in necroptosis.

Recently, with the development of PROTAC technology, targeted protein degradation has been a hopeful method for drug discovery 49-51. Notably, XD2-149, a napabucasin-based PROTAC, degrades ZFP91 with DC50 values in the nanomolar scope 20. In our study, we confirmed that ZFP91 promoted AML progression. This inspired us to envision the targeting of ZFP91 as an anti-cancer treatment strategy in AML, and the present research offers a theoretical foundation for this hypothesis.

Nevertheless, this research has not clarified the exact molecular interaction between ZFP91 and RIP1. As a novel E3 ligase, ZFP91 usually works by promoting the ubiquitination of downstream molecules. In this study, ZFP91 inhibits RIP1's ubiquitination. This result is worthy of our attention. Although the interaction of ZFP91 and RIP1 was identified by Co-IP, further study is needed to determine if this interaction is direct or indirect. We would hazard a hypothesis that ZFP91 might function as a deubiquitinase, and this might reveal its new functions that have never been studied in cells. On the other hand, we can assume that ZFP91 might inhibit the expression level or activity of some kind of E3 ubiquitin ligase, which would contribute to the ubiquitination and degradation of RIP1.

Thus, we predicted protein-protein interaction (PPI) by bioinformatics analysis. We evaluated the protein-protein interaction (PPI) networks by STRING, Genemania and HitPredict. The PPI networks and 10 key proteins interacted with ZFP91 and RIP1 were found via STRING software analysis (Fig S1A and B). The PPI networks of ZFP91 and RIP1 were conducted by Genemania network analysis (Fig S1C and D). Top 30 interacted partners with ZFP91 and RIP1 were identified by HitPredict (Table S1 and S2). Taken together, these data indicate that there may be little possibility that ZFP91 has a direct interaction with RIP1. It hints that this interaction may be mediated by another component. Nonetheless, the truly underlying mechanism warrants further investigation. Next, we will focus on the exact mechanism by which ZFP91 affects the ubiquitination of RIP1 and the further mechanism by which ZFP91 plays a role in AML.

## Conclusion

In conclusion, as we know, our data indicate that ZFP91 functions as a positive regulator of RIP1 protein stability. ZFP91 inhibits the K48-linked ubiquitination of RIP1, which contributes to leukemic cell survival and apoptosis inhibition. These outcomes offer a novel perspective of the biological function of ZFP91 and indicate that targeting ZFP91 is a promising therapeutic strategy in AML.

## Supplementary Material

Supplementary figure and tables.

## Figures and Tables

**Table 1:** Sequences of primers used for quantitative real-time PCR

Number	Gene	Forward/Reverse	Primer Sequence (5′-3′)
1	β-actin	F	TGACGTGGACATCCGCAAAG
		R	CTGGAAGGTGGACAGCGAGG
2	ZFP91	F	TGTCCTTGCCCATCCTCGCTA
		R	ACTCTTGAAGGCCCGAGCAC
3	RIP1	F	AGGCTTTGGGAAGGTGTCTC
		R	CGGAGTACTCATCTCGGCTTT
4	PCNA	F	GGCTCCATCCTCAAGAAGGTGTTG
		R	GCCAAGGTATCCGCGTTATCTTCG
5	Ki-67	F	TCCAGACACCAGACCACACTGAG
		R	GTTCTTGGCTGCCTCTTGCTACC
6	Cyclin D1	F	CCTTTGGTGCCAACTGGTGTT
		R	TCAGATGACTCTGGGAAACGC
7	Cyclin E1	F	GTGTCCTGGATGTTGACTGCCTTG
		R	GCTCTGCTTCTTACCGCTCTGTG
8	Cyclin A2	F	TCCAAGAGGACCAGGAGAATATCA
		R	TCCTCATGGTAGTCTGGTACTTCA
9	CD11b	F	CAGAGCGTGGTCCAGCTTCA
		R	CCTTCATCCGCCGAAAGTCA

## Abbreviations

AML: acute myeloid leukemia
ZFP91: ZFP91 zinc finger protein
RIP1: receptor-interacting serine/threonine-protein kinase 1
Co-IP: co-immunoprecipitation
CHX: cycloheximide
MG132: the proteasome inhibitor
PMSF: phenyl methane sulfonyl fluoride
PVDF: polyvinylidene difluoride
qRT-PCR: quantitative real-time PCR
PPI: protein-protein interaction
