# Supplementary material for: ZFP91 promotes cell proliferation and inhibits cell apoptosis in AML via inhibiting the proteasome-dependent degradation of RIP1
Source: Int J Med Sci. 2022 Jan 1;19(2):274–85. doi: 10.7150/ijms.67436 (PMC8795797; doi:10.7150/ijms.67436)
Supplement: Supplementary file 1 — Supplementary figure and tables. [file ijmsv19p0274s1.pdf]

A

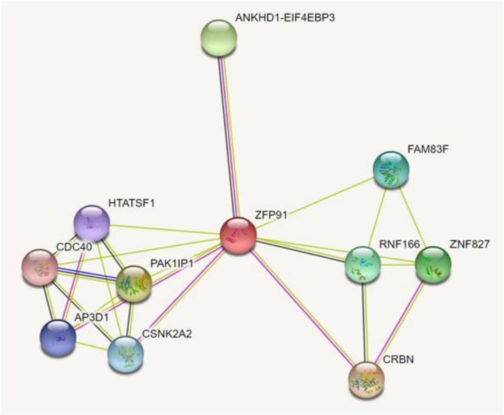

Top10 Interacted Partners:

Predicted Interacted Partners	score
CRBN	0.868
PAK1IP1	0.784
ANKHD1-EIF4EBP3	0.739
ZNF827	0.736
RNF166	0.705
FAM83F	0.686
CSNK2A2	0.673
AP3D1	0.660
HTATSF1	0.654
CDC40	0.649

B

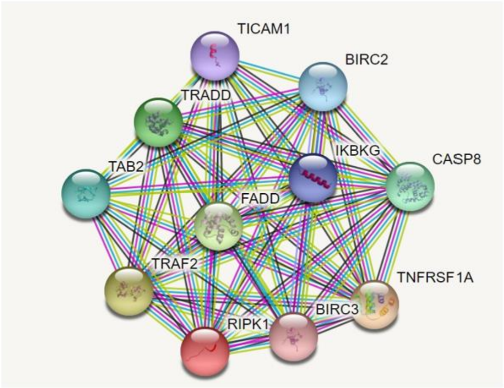

Top10 Interacted Partners:

Predicted Interacted Partners	score
TNFRSF1A	0.999
TRAF2	0.999
FADD	0.999
TRADD	0.999
CASP8	0.999
TAB2	0.999
BIRC2	0.999
IKBKG	0.999
TICAM1	0.998
BIRC3	0.998

C

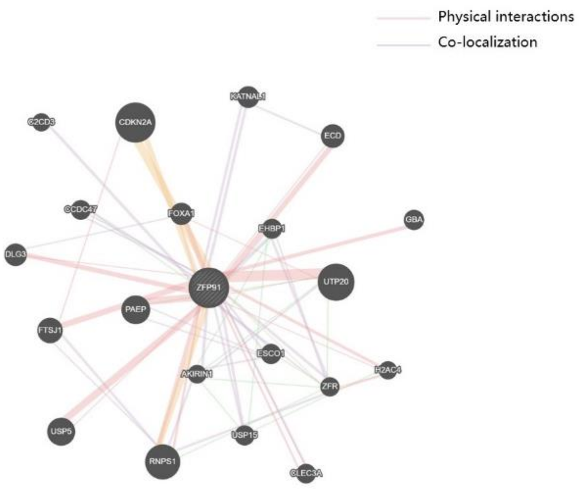

D

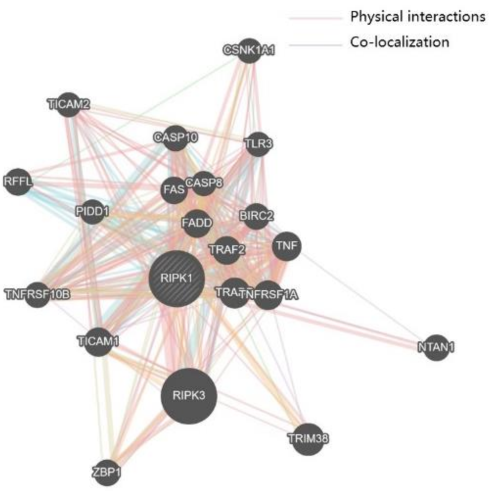

Figure S1. The PPI networks of ZFP91 and RIP1.

(A)STRING network analysis of ZFP91 protein interaction and top10 interacted partners. (B)STRING network analysis of RIP1 protein interaction and top10 interacted partners. (C)The PPI network of ZFP91 by Genemania network analysis. (D)The PPI network of RIP1 by Genemania network analysis.

Table S1: Top 30 interacted partners of ZFP91 identified by HitPredict.

Interactor Name	Experiments	Category	Method Score	Annotation Score	Interaction Score	Confidence
CRBN	3	Small-scale	0.71	1	0.84	High
M3K14	1	Small-scale	0.56	1	0.747	High
PRDM5	1	High-throughput	0.48	1	0.691	High
SRPK2	1	High-throughput	0.42	1	0.647	High
ZFP41	1	High-throughput	0.39	1	0.621	High
ZN526	1	High-throughput	0.39	1	0.621	High
FBRL	2	High-throughput	0.62	0.6	0.611	High
FOXA1	2	Small-scale	0.57	0.6	0.584	High
ESR1	2	High-throughput	0.54	0.6	0.571	High
KAP0	2	High-throughput	0.54	0.6	0.571	High
KI67	1	High-throughput	0.48	0.6	0.536	High
CTR9	1	High-throughput	0.48	0.6	0.536	High
BHA15	1	High-throughput	0.48	0.6	0.536	High
TSYL5	1	High-throughput	0.48	0.6	0.536	High
CHC10	1	High-throughput	0.48	0.6	0.536	High
AKIP	1	High-throughput	0.48	0.55	0.513	High
CLC3A	1	High-throughput	0.48	0.5	0.489	High
TRM7	1	High-throughput	0.48	0.5	0.489	High
PO6F2	1	High-throughput	0.39	0.6	0.481	High
ASPP2	1	High-throughput	0.39	0.6	0.481	High
FAM9A	1	High-throughput	0.39	0.6	0.481	High
TSYL2	1	High-throughput	0.39	0.6	0.481	High
PICK1	1	High-throughput	0.39	0.6	0.481	High
BRD4	1	High-throughput	0.38	0.6	0.479	High
RECQ4	1	High-throughput	0.38	0.6	0.479	High
FOS	1	High-throughput	0.38	0.6	0.479	High
P53	1	High-throughput	0.38	0.6	0.479	High
H2A1B	1	High-throughput	0.38	0.6	0.479	High
APC	1	Small-scale	0.38	0.6	0.479	High
BRD2	1	High-throughput	0.38	0.6	0.479	High

Table S2: Top 30 interacted partners of RIP1 by identified HitPredict.

Interactor Name	Experiments	Category	Method Score	Annotation Score	Interaction Score	Confidence
TNR1A	43	Small-scale	0.99	1	0.994	High
FADD	25	Small-scale	0.98	1	0.989	High
RIPK3	19	Small-scale	0.98	1	0.989	High
BIRC2	10	Small-scale	0.96	1	0.981	High
RIPK1	9	Small-scale	0.95	1	0.975	High
TRADD	7	Small-scale	0.93	1	0.964	High
BIRC3	5	Small-scale	0.88	1	0.938	High
M3K7	4	Small-scale	0.76	1	0.873	High
TCAM1	4	Small-scale	0.76	1	0.873	High
TR10B	3	Small-scale	0.68	1	0.827	High
XIAP	2	Small-scale	0.62	1	0.785	High
CRADD	2	Small-scale	0.6	1	0.778	High
CASP8	37	Small-scale	0.99	0.6	0.77	High
NEMO	25	Small-scale	0.98	0.6	0.767	High
TRAF2	23	Small-scale	0.98	0.6	0.767	High
TNFA	24	Small-scale	0.98	0.6	0.765	High
PIDD1	2	Small-scale	0.57	1	0.754	High
TNAP3	7	Small-scale	0.93	0.6	0.746	High
CSK21	2	High-throughput	0.54	1	0.737	High
CSK23	2	High-throughput	0.54	1	0.737	High
KC1A	1	Small-scale	0.54	1	0.733	High
TRAF1	5	Small-scale	0.87	0.6	0.722	High
CFLAR	5	Small-scale	0.84	0.6	0.709	High
MRCKB	1	High-throughput	0.49	1	0.698	High
OPTN	4	Small-scale	0.8	0.6	0.692	High
CYLD	4	Small-scale	0.76	0.6	0.676	High
TAB2	4	Small-scale	0.76	0.6	0.676	High
LRRK2	1	Small-scale	0.44	1	0.665	High
ASC	1	High-throughput	0.44	1	0.665	High
TRAF3	3	Small-scale	0.73	0.6	0.664	High
